# The Prognostic Significance of Small Size in Breast Cancer

**DOI:** 10.1038/bjc.1953.4

**Published:** 1953-03

**Authors:** L. Kreyberg, T. Christiansen

## Abstract

**Images:**


### THE PROGNOSTIC SIGNIFICANCE OF SMALL SIZE IN BREAST CANCER.

L. KREYBERG AND T. CHRISTIANSEN.

*From the Institutt for Generell og Eksperimentell Patologi, Universitetet i Oslo.*

Received for publication February 2, 1953.

IT is a strange fact that in the voluminous literature on breast cancer the prognostic value of the size of the tumour is only cursorily dealt with, a fact mentioned by Geschickter (1945) and underlined by Bloom (1950). The explanation probably is, that the view expressed by Kaae (1948), namely, " It is generally recognized that the size of the tumour is a very important factor in the prognosis, and that the latter is much more favourable for the small tumours than for the large," seems so generally sound, even self evident, that the problem has not been considered worthy of a closer study.

If we examine the pertinent literature, we find, however, that the findings are not unequivocal.

Dahl Iversen (1930) states that breast cancers up to the size of plums are without recurrences after 3 years' observation in 83 per cent of the cases, whereas tumours larger, up to the size of a hen's egg, show only 13 per cent freedom from recurrences after 3 years. Eggers, de Cholnoky and Jessup (1941) report upon a 5-year survival of 73 per cent, if the mass is 2 cm. or less, and only 24 per cent if the mass has reached a size of 3 to 6 cm. Haagensen and Stout (1943) found a 5-year clinical cure of 62·2 per cent if the tumour was under 3 cm. and only 19·8 per cent if the tumour had reached 6 cm. or more. They conclude that " The data indicate, as might be expected, that the prognosis becomes worse as the size of the tumour increases ".

Bloom (1950) found 5-year survivals in the following percentages : tumour less than 1 inch 59 per cent, 1 to 2 inches 45 per cent, and more than 2 inches 32 per cent, and he finds after grading of the tumours that : " Of the tumours with a diameter of 1 inch or less, 37 per cent are classified as Grade I and 23 per cent as Grade III. On the other hand, in the case of growths of more than 2 inches diameter only 8 per cent belong to Grade I whilst 54 per cent are Grade III. When the diameter lies between 1 and 2 inches the incidence of these tumours is practically the same ", and he concludes that : " Whether the tumours are small or large, the outlook is uniformly good in the former (Grade I) and bad in the latter group (Grade III). On the other hand, an intermediate result is obtained for the intermediate cases (Grade II), the survival rate being practically halved in the presence of the larger neoplasms. In other words, the metastasizing power for Grade I and also Grade III cancers is independent of size. For growths classified as Grade II this power bears a direct relationship to the diameter, the larger the tumour the greater the likelihood of spread having taken place ". Most probably this holds good also for the Grade I and Grade III tumours, only that the time factors are so long or so short that their significance is obscured under the usual clinical conditions.

When Kunath (1940) says: "*Size and mass.*—An analysis of this point failed to reveal that a larger tumour carries any more serious a prognosis than does a small one", and Hoopes and McGraw (1942) likewise conclude: "No correlation was found between the post-operative duration of life and the size of the tumour removed", these findings are not necessarily in contradiction to the opposite conclusions, mentioned above. The explanation may be a different composition of the groups of tumours, as regard number of representatives of the different grades of malignancy. Again we have to guard ourselves in transferring conclusions from groups to individual cases.

Considering the importance of assessing the significance of a minimum size of tumour for the prognosis of breast cancer, we have examined breast cancer material consisting of a total of 974 cases. We planned to examine the ultimate development of breast cancers as small as practically diagnosable. In order to decide the upper limit of the size to include, theoretical and practical considerations have been taken into account. Firstly, we considered the smallest lump, distinguishable as a definite tumour. That size evidently depends upon the site and upon the amount of adipose tissue. A tumour near the surface and in a shrunken atrophic breast will be more easily discovered than one deeply situated in a full and developed breast. It seems that tumours smaller than those the size of a pea cannot reasonably be distinguished from the many irregular nodosities in a climacteric, or pre-climacteric breast, and in most cases it is difficult to feel a lump smaller than a small hazel nut. Secondly, we wanted to examine tumours of a size so small that the number of cases would represent a very small fraction of the total, whereby we would obtain a statistical claim to a designation "a very small breast tumour". The size of our tumours is not given in centimetres, or millimetres. The tumours are usually characterized by some object for comparison, usually: pea, bean, nut kernel, hazel nut, date-stone, date, wall nut, plum, etc. A tumour the size of a hazel nut will usually measure some 10 to 12 by 15 to 17 mm. One has to have in mind the small size of a Norwegian hazel nut to appreciate the size.

When we examined our total material we found that only 56 tumours out of 974, i.e., 5·7 per cent. had the size of a hazel nut, or were smaller, and only 10 tumours (1 per cent) were recorded as large as a pea, or a bean. This indicates that we have found a suitable standard, covering our demand for a diagnosis of a breast tumour as small as practically diagnosable. If smaller nodules should be removed for diagnostic or prophylactic purposes, the number of amazons would reach such proportions that a general reaction from the population would be the result.

Our conclusion is further substantiated by the previous papers dealing with this problem. Table I shows the standards and the relative occurrence of the smallest groups recorded:

TABLE I.—*The Maximum Size of the Breast Tumours and the Relative Occurrence of the Size Mentioned.*

| | cm. | |
|---|---|---|
| Eggers, Cholnoky and Jessup (1941) | 1 | 6 per cent of all. |
| | 2 | 15 ,, ,, |
| Bloom (1950) | 2·5 (1 in.) | 50 ,, ,, |
| Haagensen and Stout (1943) | 3 | 15 ,, ,, |

The present material consists of breasts and lymph nodes received for diagnosis from city and county hospitals, as well as from private surgeons, during the years 1933 to 1942. It is representative, and comparable to any other large, average similar material in Scandinavia. It should comprise cases with at least 75 per cent operability and include a fair number of " early " cases, as the population is rather cancer conscious, being under a constant barrage of cancer propaganda with promises of great hopes of cure if presenting themselves for treatment at an early stage. The fate of the patients has been followed for from 10 to 20 years. The material was examined during the later half of 1952.

The clinical information is not always precise, or detailed, as some of the case histories are very short. Nor is the pathological material always complete, as some of the surgeons do not submit adipose tissue from the axilla if no suspicious glands are found. In other cases a selection of glands only are sent to the laboratory, and finally, some of our paraffin blocks were destroyed during the war, with the result that for some cases a few slides only could be examined. This means that all the positive findings are minimum recordings.

The clinical follow-up has been complete, all patients having been accounted for. Reliable death certificates have been received for all the dead, and the patients alive have been personally examined by one of us, or by competent doctors as regards the patients living in remote parts of the country.

As only the patients with a definite statement as to the *size of the tumour* have been included, the size is known in all cases. The designations have been: slightly larger than a hazel nut, 10 cases ; plum stone, 1 case ; half a wall nut, 1 case ; hazel nut, 22 cases ; nut kernel, 11 cases ; pea nut, 1 case ; pea, or bean, 10 cases.

*Grade of malignancy* of the primary tumour, 11 cases were recorded as Grade I, 28 cases as Grade II, and 17 cases as Grade III.

*Axillary lymph nodes* were submitted for examination in 31 cases, lymph nodes not examined in 25 cases.

*Treatment* varied. In 7 cases a local operation only, and in 7 cases radical operation was performed. In all the other cases the operation was combined with radiological treatment, before, or after the operation, or both. In some cases X-rays were used, in other cases tele-radium. As information is sparse, details are omitted. No conclusions will be drawn as to the methods of treatment, other than to state that the patients have received the varied treatment offered the average breast cancer patient in Norway.

*The duration of symptoms*, that is, the interval between the first recorded symptom until treatment was commenced, was recorded in 41 cases.

*The period of survival* has been calculated from the commencement of treatment up to the death of the patient, or the day of clinical examination during 1952.

### RESULTS.

In Table II is recorded the findings as regards the question of symptom-free survival for the 5 years' and the 10 years' observation.

TABLE II.—*Total Number* (56 *Cases*) ; *Alive, Symptom-free.*

| | | |
|---|---|---|
| After 5 years | 37 | 66 per cent |
| After 10 years | 36 | 64 ,, |

In this table all dead during the period are without reservation, counted as dead from breast cancer. No 2 died 14 days after operation, when leaving the hospital. No. 43 died a sudden death 6 months after operation, and No. 8 died from broncho-pneumonia one year and a half after operation, symptom-free. All the others succumbed to their tumours.

This 5-year cure rate corresponds very closely to the rate quoted by Engelstad (1948) for his group of 768 cases, regarded as Stage I and Stage II cases. This indicates that our " small " breast cancers do not present a particularly favourable clinical picture.

In one aspect our Table II shows a remarkable deviation from the usual, namely, the small difference between the 5-year and the 10-year figures, only 1 out of 37 patients dying in the second 5-year interval.

As stated, the whole group of 56 patients has been followed for 10 years. A smaller group has, however, been followed for a longer period, and in Table III some of the results are summarized. The 11 years' observation group ends by the material of the 1941 group, the 12 years' observation material ends by the 1940 group and so on. As therefore the material is not identical in all groups, it is evident that the results may present features which may look inconsistent at the first impression. The findings for the 11th to 14th years are given in Table III.

TABLE III.—*Alive, Symptom-free.*

| | | | |
|---|---|---|---|
| After 11 years | 31 out of 48 | 65 per cent | (No. 27 died symptom-free. No. 28 and 29 with metastases, dying next year.) |
| „ 12 „ | 24 „ 40 | 60 „ | (No. 10 died symptom-free. No. 30 with metastases, dying next year.) |
| „ 13 „ | 15 „ 35 | 43 „ | (No. 31 died symptom-free. No. 20, 24, 32 with metastases, dying next year.) |
| „ 14 „ | 8 „ 17 | 47 „ | — |

Table III shows the very important fact that a considerable number of patients arrive at the 10 years' limit in apparently good condition, but that nevertheless they are bearers of silent metastases, which eventually terminate their lives.

As 17 patients only, out of the total of 56, have been followed for as long as 14 years, a number of fatal metastases are probably due among the remaining, and we may conclude that more than half of all the patients presenting themselves with a tumour, the size of a small hazel nut, or smaller, are potential victims of

---

EXPLANATION OF PLATE.

FIG. 1.—G. N—, 61 years old. J. No. 280/38. Carcinoma Grade I. Size: hazel nut. Symptoms two weeks. Radical operation. Post-operative radium treatment. 14 years' observation. Symptom-free. × 135.

FIG. 2.—W—, 48 years old. J. No. 2507/40. Carcinoma Grade I. Size: hazel nut. " Brief " case history. Radical operation. Post-operative X-rays. Symptom-free, 12 years' observation. × 135.

FIG. 3.—M. E—, 55 years old. J. No. 1721/40. Carcinoma Grade I. Size: bean. Unknown duration of symptoms. Radical operation. Post-operative X-rays. Symptom free, 12 years' observation. × 135.

FIG. 4.—L. S—, 60 years old. J. No. 554/40. Carcinoma Grade III. Size: hazel nut. Symptoms a few days. Radical operation. Three lymph nodes and perinodular adipose tissue infiltrated by tumour cells. The picture is from one of the lymph nodes. Post-operative X-rays. Symptom-free, 12¾ years' observation. × 155.

1 2 3 4

Kreyberg and Christiansen.

their cancer, when given the average treatment in use in Norway. Even if we select the very smallest tumours present in our series, those the size of a pea or a bean, out of a total of 10, we find only 5 patients alive after 10 years, or more, and 4 patients dead from metastases.

In the present series the combined surgical and radiological treatment seems, in a number of cases, to have effected a considerable delay in a finally fatal outcome. This delay is of very great value from a therapeutic standpoint, even if the treatment fails to effect a complete cure.

The prolonged observation also helps to assess more accurately the real stage of the tumours. Staging actually is an ambiguous process. The simplest and least reliable staging is the immediate clinical, unreliable to a degree to make it practically useless. The anatomical staging is somewhat more precise, and, in lack of anything better, the accepted basis for grouping of tumours for comparison of therapeutic results. We have, however, to bear in mind that the value even of this grouping is rather limited. Negative findings of tumour cells in lymph nodes removed may be the result of the pathologist not finding the cells in spite of their presence. Or, tumour cells may by-pass the usual lymphatic filters in the axillary glands and proceed directly to the supraclavicular or the intrathoracic nodes, or the tumour cells may spread via the blood stream. The discrepancy between the number of tumours anatomically staged as Stage I and the number of cures effected after a proper local operation shows the degree of failure in the process of staging. The average failure is generally at least 20 per cent. The final and most accurate staging is a retrospective process, taking into consideration the outcome of the cancerous growth. A number of patients staged as Stage I eventually through the clinical development are shown to be in Stage II, or further. On the other hand, if radiological treatment has been added to the surgical, even the delayed retrospective clinical staging may be incomplete, because a tumour, anatomically staged as Stage I and actually being in Stage II, may be cured by radiological treatment and thereby escape its proper staging.

In spite of the incomplete clinical and pathological information, it will be of some interest to examine the state of affairs, as regards stages recorded in this material. The data available are shown in Table IV.

TABLE IV.—*Stages : Status by the end of* 1952.

| | Alive, symptom-free. Minimum 10 years. | Dead, and patients living with metastases. |
|---|---|---|
| Lymph nodes negative | 13 | 7 |
| One lymph node invaded | 3 } 5 | 1 } 6 |
| Several lymph nodes invaded | 2 } | 5 } |
| State of lymph nodes unknown | 9 | 16* |
| Total | 27 | 29 |

* No. 10, 27, 31 died symptom-free after more than 10 years' observation.

The large number of fatal outcomes with negative findings of tumour cells in the axillary lymph nodes confirm our assumption that our figures represent minimum findings. The considerable number of patients alive and symptom-free in spite of positive findings of tumour cells in the lymph nodes, actually 5 patients, further confirm our statement as to a considerable effect of the treatment, but also underlines the great number of patients already in Stage II (or further) at the moment of presenting a very small breast cancer.

If we add these 5 patients with microscopical lymph-node metastases, but still symptom-free, to the group of the dead and those living with metastases, we find that two-thirds of all our patients with small tumours were in Stage II (or further) when the operation was performed.

The value of Bloom's (1950) grading and analysis of his material made it natural to try his principles on our material. We deliberately use the word principles, because any grading is arbitrary, and the designation in each case is the result of a subjective estimate of a series of characteristics, which themselves are unprecise. With this reservation, which possibly will be further substantiated by our findings, we present our material in Table V.

TABLE V.—*Grades : Status of our Material by the end of* 1952.

| | Alive, symptom-free. Minimum 10 years. | Dead, or patient living with metastases (known). |
|---|---|---|
| Grade I | 11 | 0 |
| „ II | 8 } 15 | 20* } 30 |
| „ III | 7 | 10 |
| Total | 26 | 30 |

* No. 10, 27, 31 died symptom free after more than 10 years' observation.

This survey confirms the importance of recognizing and considering the Grade I cases when statistics are prepared of breast cancer materials. All our Grade I patients, a total of 11, and 20 per cent of the whole group, are alive, one of them having presented tumour cells in an axillary lymph node. It may be pertinent to note that all these tumours designated as Grade I are histologically definite carcinomas, not merely cases of " atypical proliferation ", " carcinoma *in situ* ", or similar conditions (Fig. 1, 2, 3). On the other hand, however, we have not succeeded in registering clinical evidence of an increasing malignancy with the passing from Grade II to Grade III. The explanation may be that the number of cases is too small, that the treatment given may have influenced the final result because a number of very malignant tumours are comparatively radio-sensitive, or it may be caused by our incompetency. We reproduce the histological picture of one of our most malignant tumours (Case No. 36), recorded as Grade III and with 3 lymph nodes invaded. The patient is still alive and feeling well nearly 13 years after the diagnosis was made (Fig. 4). She had radical mastectomy, and afterwards was given a series of X-ray treatments at one of our county hospitals (Telemark Fylkessykehus). This case, as well as some others, show that in individual cases all hope shall not be given up, even if the situation looks very dark.

If we return to our Grade I cases, we find that these tumours represent a little more than 40 per cent of all the patients alive and symptom-free. If we therefore deduct these tumours from the material assembled in Tables II and III we find a considerably less favourable result, as shown in Table VI.

Table VI shows that if we exclude the Grade I tumours, which actually have a very low malignancy, approximately two-thirds of the patients in our series have died or will die from their breast cancers.

TABLE VI.—*Alive and Symptom-free. All Cases, less Grade I.*

| Years. | | Per cent. |
|---|---|---|
| 5 | 26 out of 45 | 58 |
| 10 | 25 „ 45 | 56 |
| 11 | 20 „ 37 | 54 |
| 12 | 14 „ 30 | 47 |
| 13 | 8 „ 28 | 29 |
| 14 | 5 „ 14 | 36 |

In Table VII are collected the data as regards duration of symptoms and prognosis.

TABLE VII.—*Duration of Symptoms and Prognosis.*

| | 4 weeks. | 2–3 months. | 4–6 months. | 7–12 months. | More than 1 year. |
|---|---|---|---|---|---|
| Alive, symptom-free | 10 (4) | 4 | 2 (2) | 1 | 5 (3) |
| Dead, or alive with metastases | 6 | 3 | 2 | 6 | 2 |

This table does not include all cases, but only those where the duration of symptoms before treatment is known. Grade I cases in brackets.

At a first sight there seem to be two comparatively favourable periods: a relatively good prognosis for the patients with a very short (4 weeks) and for those with a very long (more than a year) history of symptoms. The medium long histories, from a few months up to a year, show an increasingly gloomy picture.

If we again deduct the Grade I cases we conserve the pattern, and still find indications of a benefit for very quick reaction with immediate treatment after diagnosis, and with a still more pronounced bad prognosis in cases with delays from a few months up to a year. For the very long histories (more than a year) the comparatively better prognosis is preserved, and may partly be accounted for by a default in the process of grading, partly by combination of high malignancy and high grade radiosensitivity in certain cases, and partly by inclusion of tumours with a more moderate malignancy (Grade II). Besides, a perfect correspondence between morphological grading and biological development is evidently not obtainable. Too many unknown factors are involved here. To elucidate this question a larger material is necessary.

### SUMMARY AND CONCLUSIONS.

The present study of the fate of patients presenting mammary carcinomas the size of a hazel nut, or smaller, shows that one half of the group have eventually succumbed to their tumour. If we deduct the Grade I tumours, one-third only of the patients survives. At the commencement of treatment two-thirds of the patients are in Stage II, or further.

The combined surgical and radiological treatment seems to have effected a considerable delay in the progress of the cancerous growth, and in some cases to have resulted in a complete cure, even when axillary metastases were present. The delayed development of the tumours is manifested by an insignificant difference in the 5 years (66 per cent) and the 10 years (64 per cent) figures for "alive symptom-free" and by the occurrence of a significant number of metastases after the tenth year of observation after treatment.

*Conclusion.*—The small malignant mammary tumours give, as a group, not decidedly, or appreciably, more favourable prognosis than an average group of breast cancers belonging to the ordinary average group of Stage I and Stage II cases undergoing the same type of treatment.

### REFERENCES.

BLOOM, H. J. G.—(1950) *Brit. J. Cancer*, **4**, 347.
DAHL IVERSEN, E.—(1930) *Ugeskrift for Laeger*, **92**, 1090.
EGGERS, C., DE CHOLNOKY, T., AND JESSUP, D. S. D.—(1941) *Ann. Surg.*, **113**, 321.
ENGELSTAD, R. BULL.—(1948) *Amer. J. Roentgenol.*, **60**, 776.
GESCHICKTER, C. F.—(1945) 'Diseases of the Breast', 2nd ed. Philadelphia (Lippincott). p. 403.
HAAGENSEN, C. D., AND STOUT, A. P.—(1943) *Ann. Surg.*, **118**, 859.
HOOPES, B. F., AND MCGRAW, A. B.—(1942) *Surgery*, **12**, 892.
KAAE, S.—(1948) *Acta radiol., Stockh.*, **29**, 475.
KUNATH, C. A.—(1940) *Arch. Surg.*, **41**, 66.


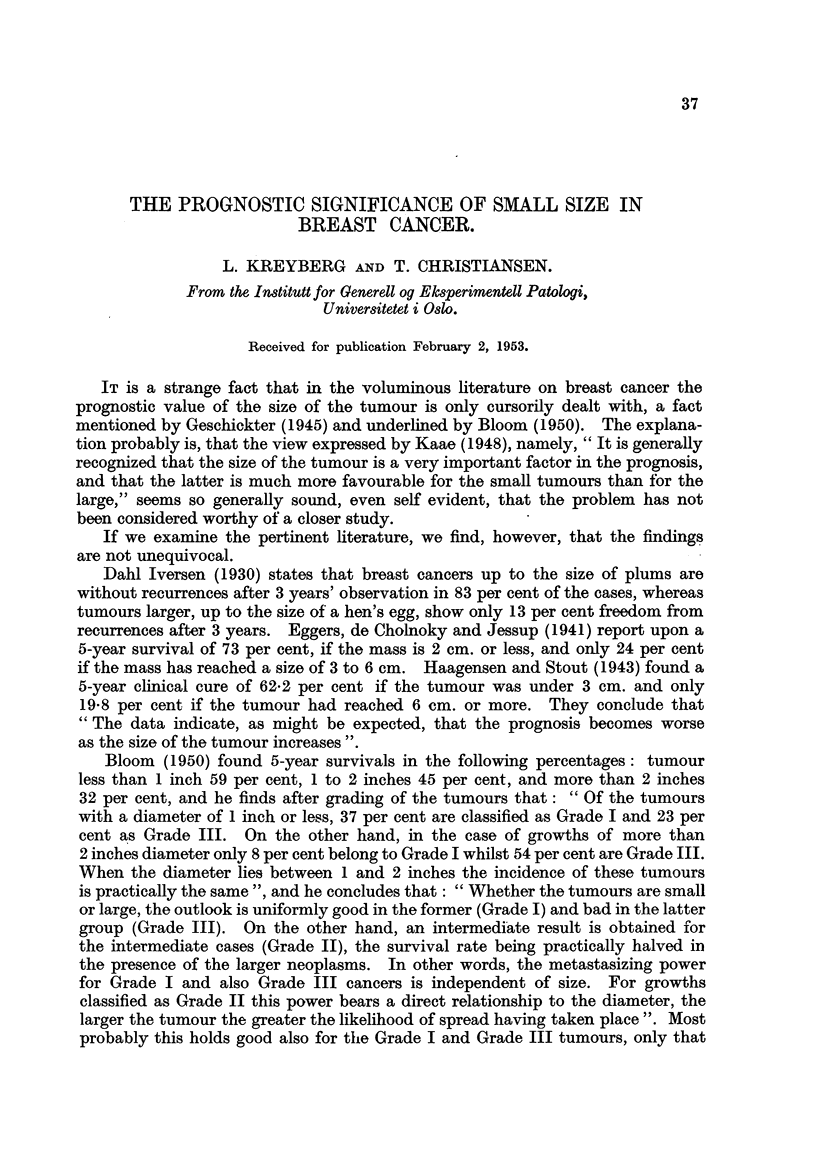

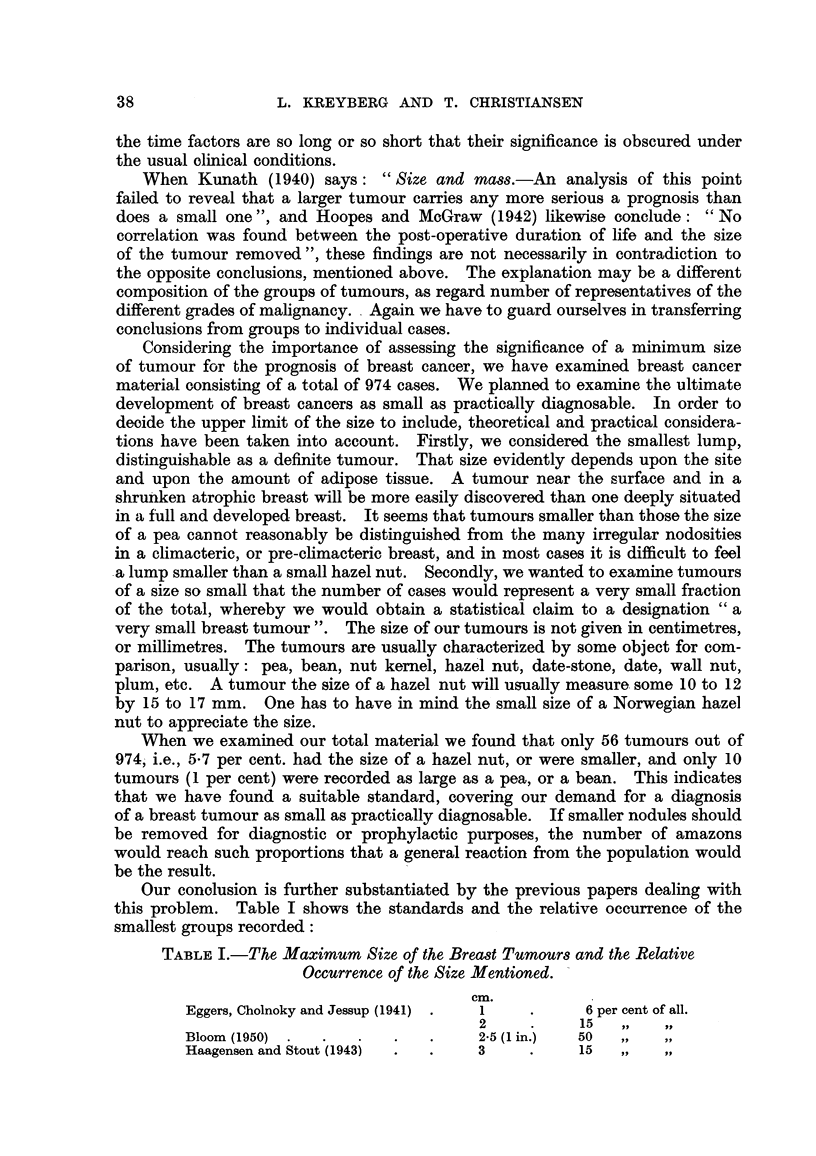

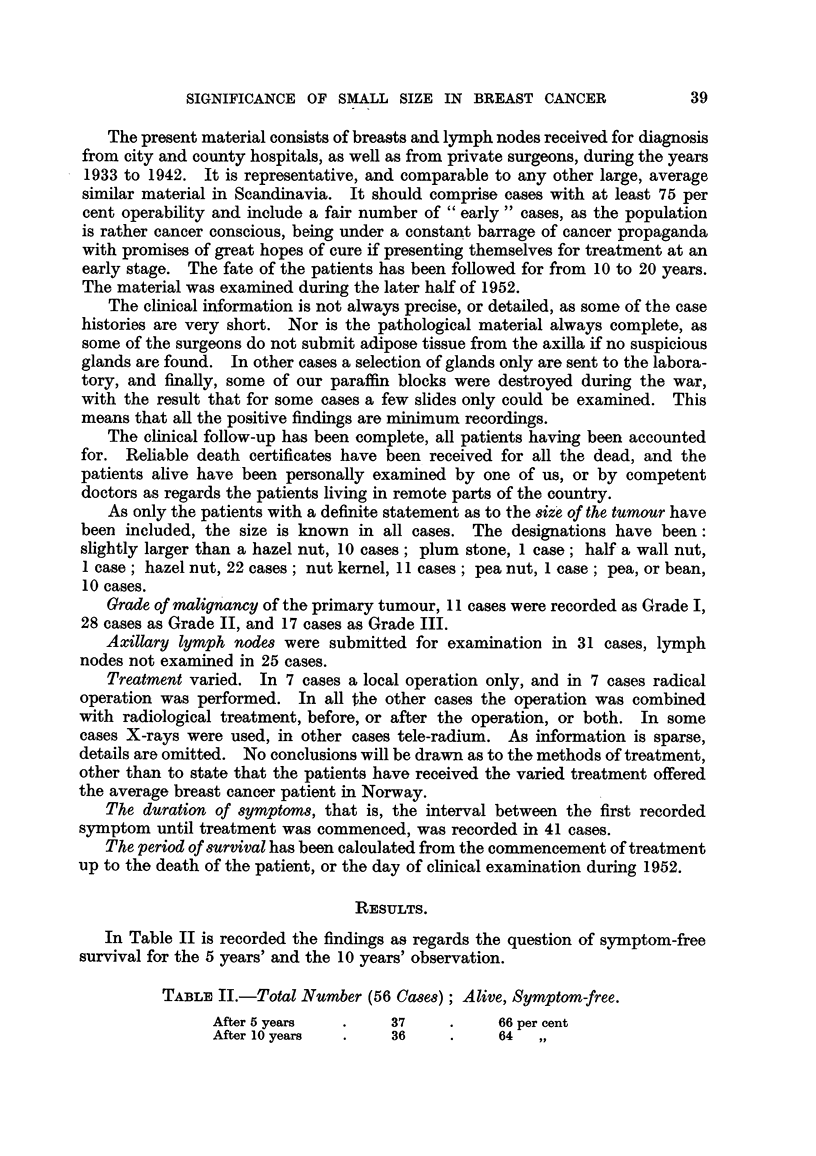

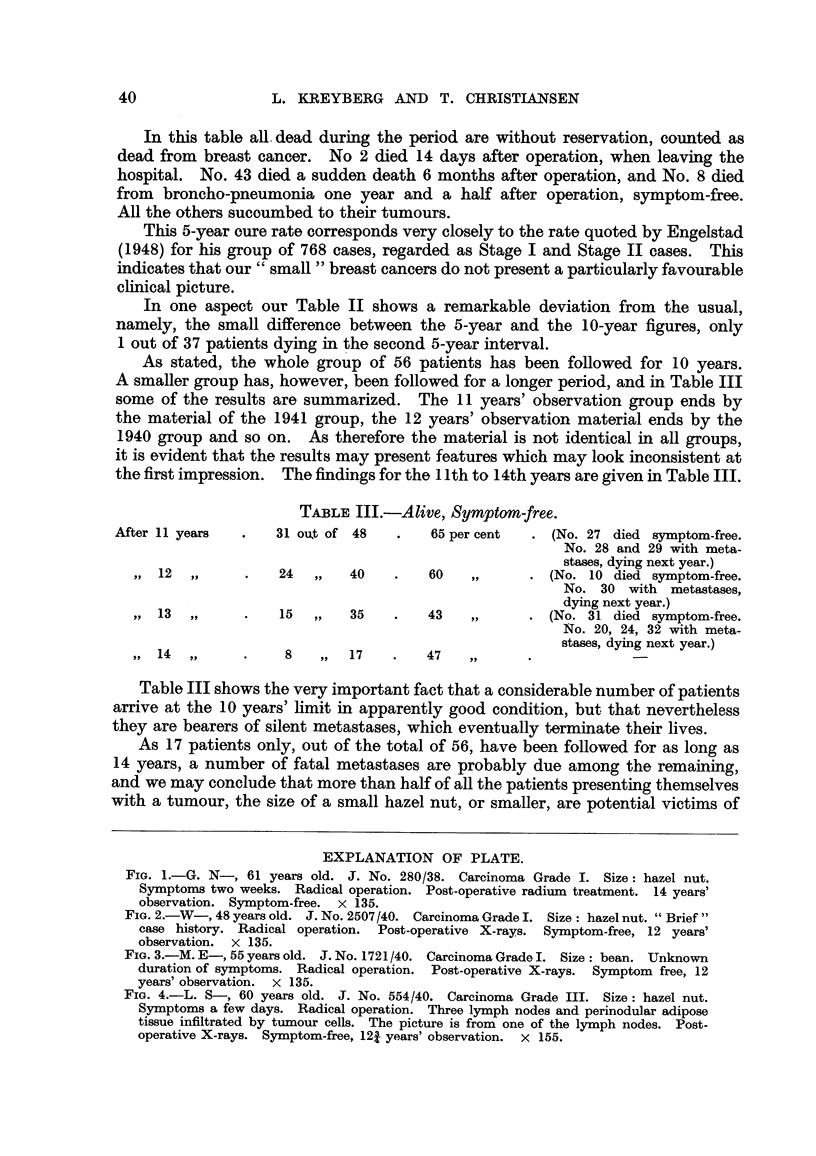

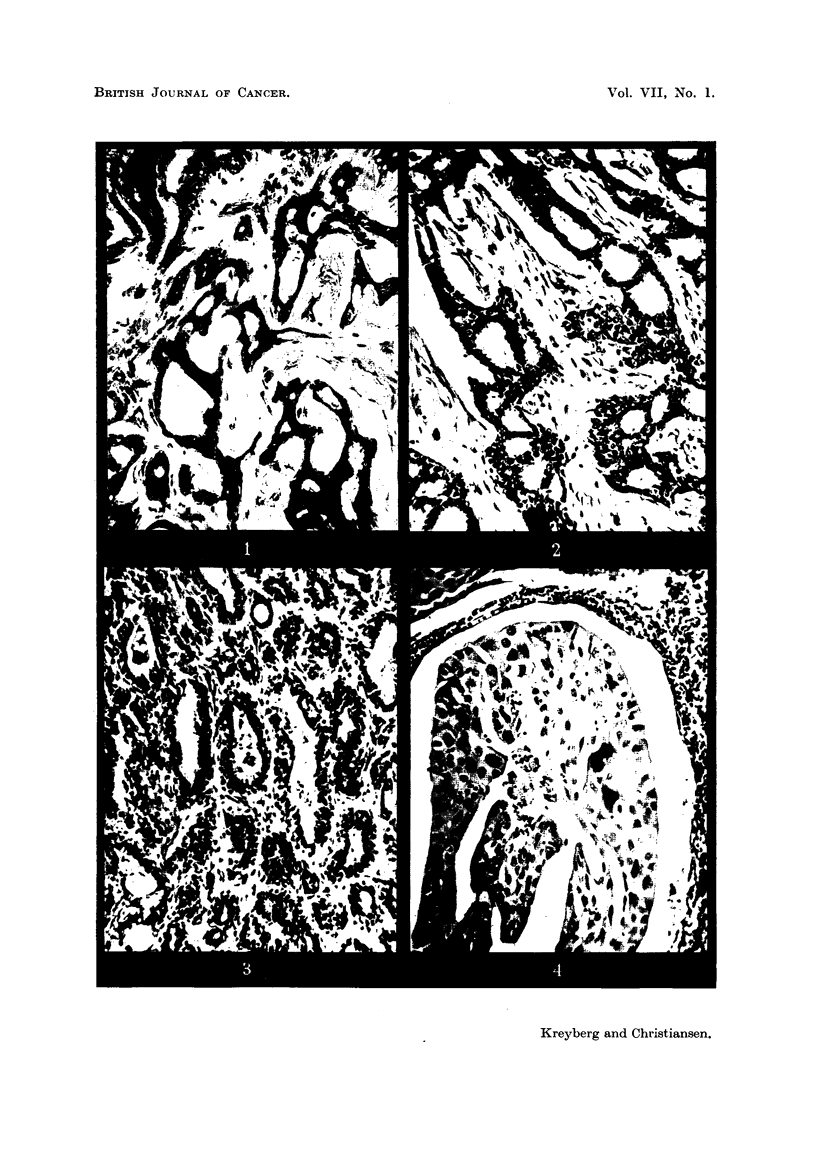

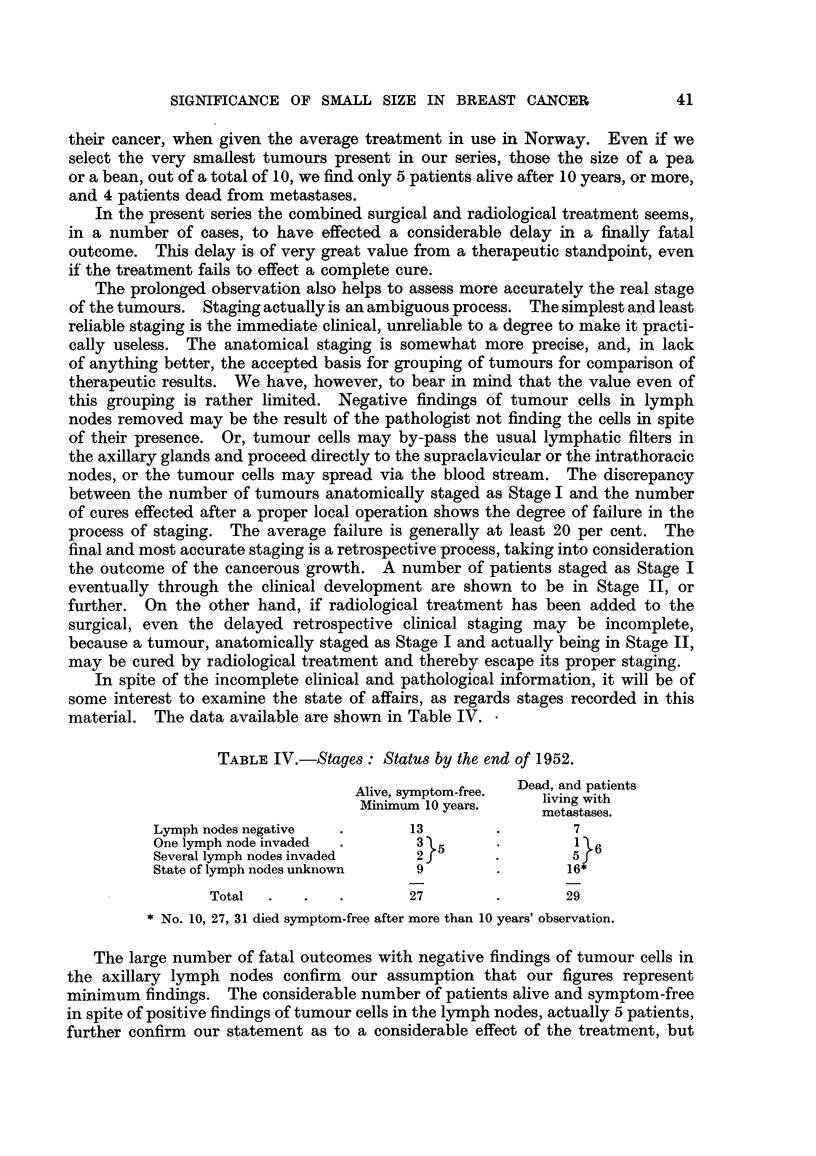

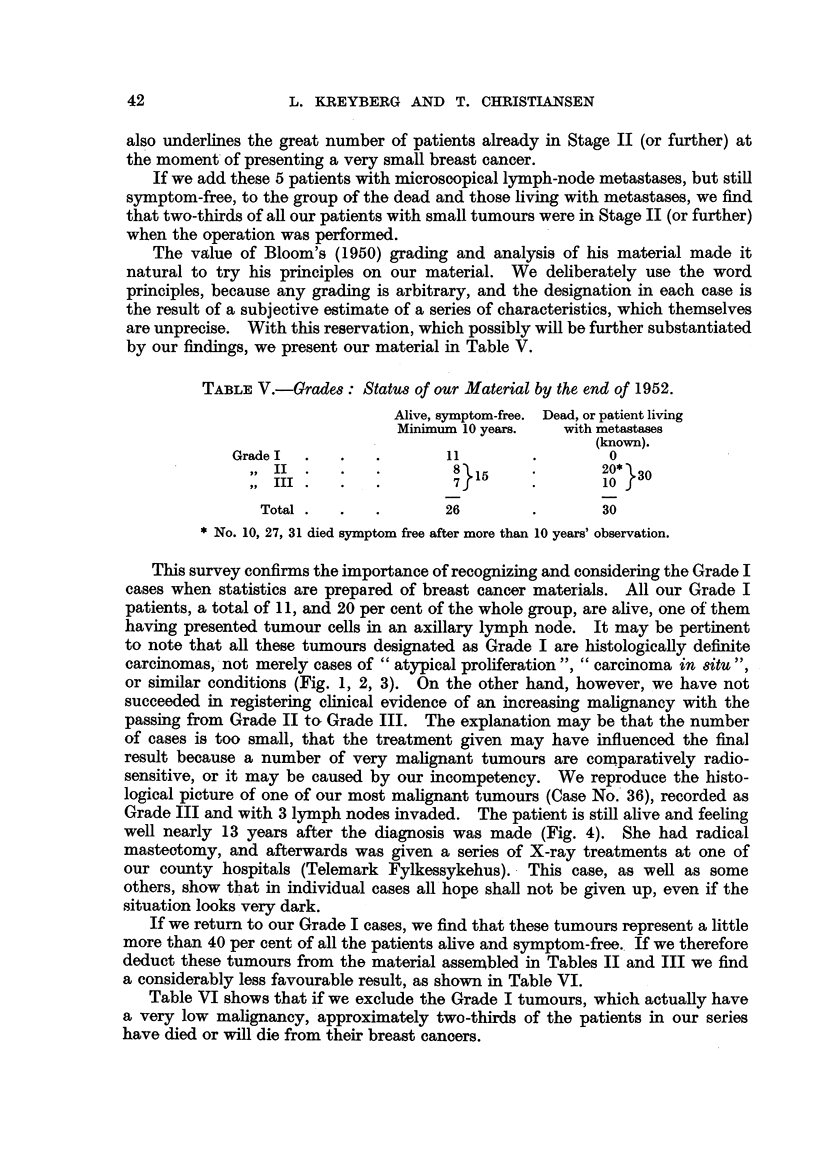

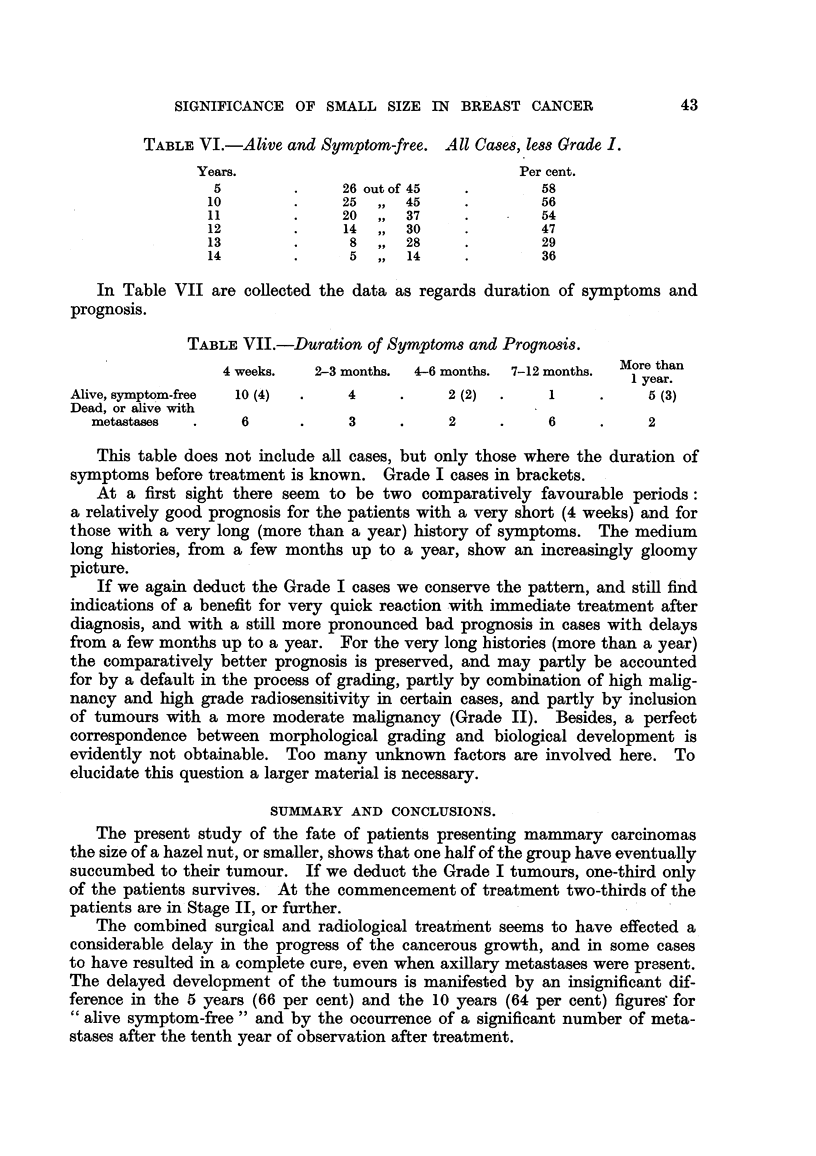

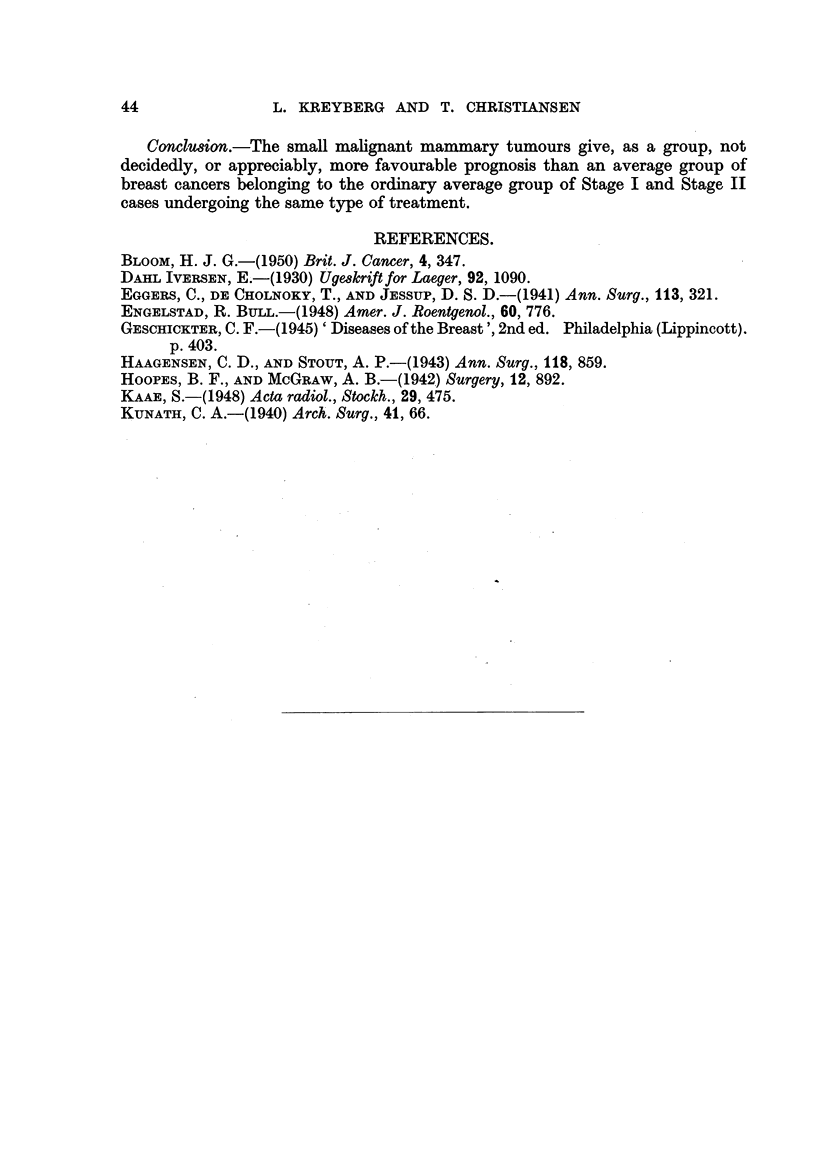


## References

[OCR_00484] BLOOM H. J. G. (1950). Further studies on prognosis of breast carcinoma.. Br J Cancer.

[OCR_00488] Eggers C., De Cholnoky T., Jessup D. S. (1941). CANCER OF THE BREAST.. Ann Surg.

[OCR_00495] Haagensen C. D., Stout A. P. (1943). CARCINOMA OF THE BREAST: II. CRITERIA OF OPERABILITY.. Ann Surg.

